# Supplementary figures and images for: The geography and inter-community configuration of new sexual partnership formation in a rural South African population over fourteen years (2003–2016)
Source: PLOS Glob Public Health. 2022 Mar 9;2(3):e0000055. doi: 10.1371/journal.pgph.0000055 (PMC10022024; doi:10.1371/journal.pgph.0000055)

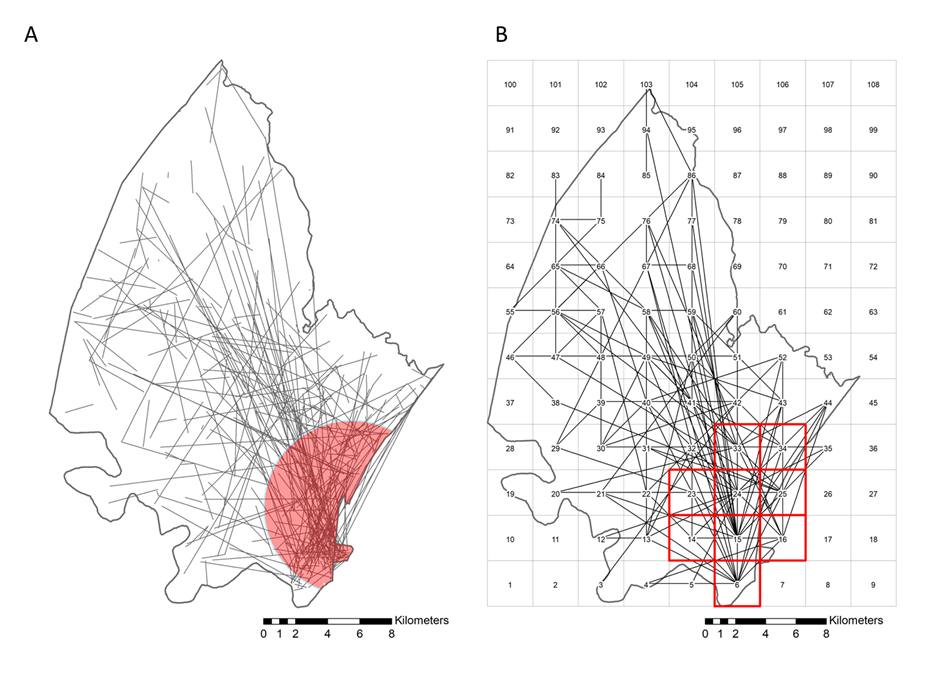

Supplement: S1 Fig — Links generated by the formation of the stable sexual partnerships among individuals located within the surveillance area (A), where aggregated in nodes formed by the centroids of the pixels generated by a 3km x 3km grid that covered the entire surveillance area (B). Cells delineated in red illustrate are located within the peri-urban cluster area (red area in A). Nodes were labeled using sequential numbers. (TIF) [file pgph.0000055.s001.tif]

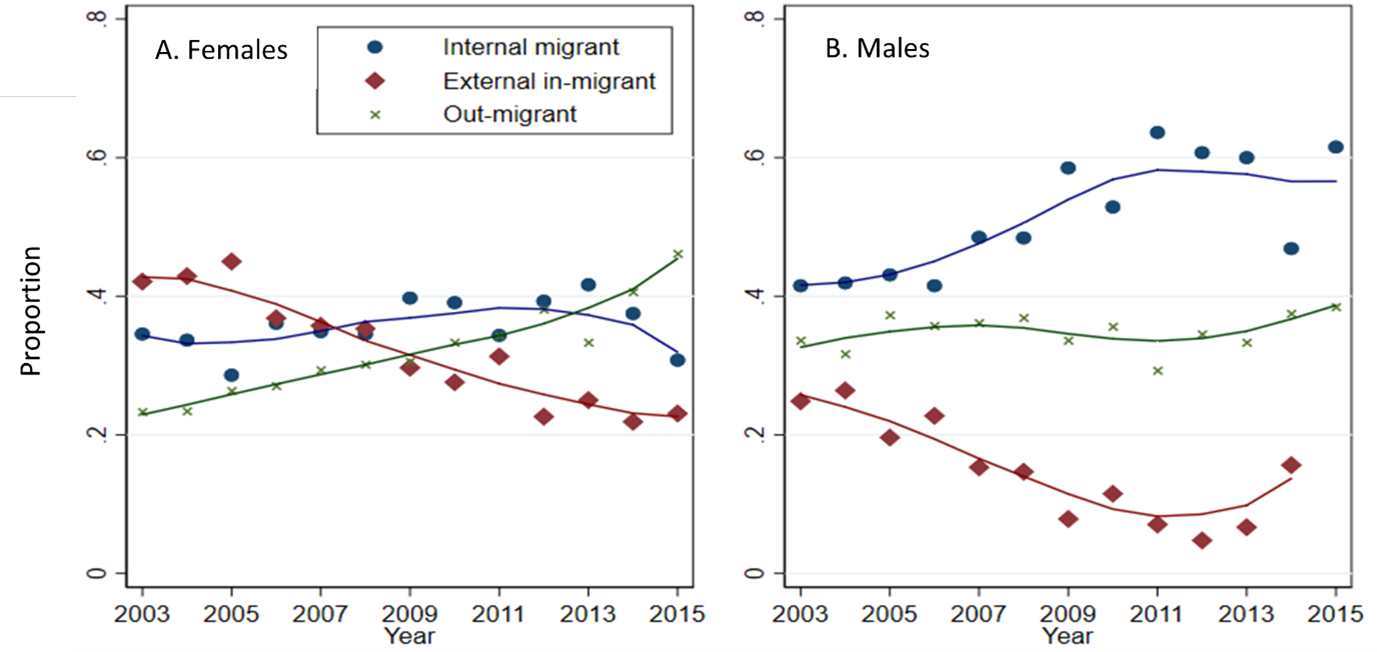

Supplement: S2 Fig — Types of migration in sexual partnership formation by gender in 2003–2015: (A) Females and (B) Males. Blue circle, red diamond, and green cross symbols represent internal migrants, external in-migrants, and out-migrants, respectively. (TIF) [file pgph.0000055.s002.tif]

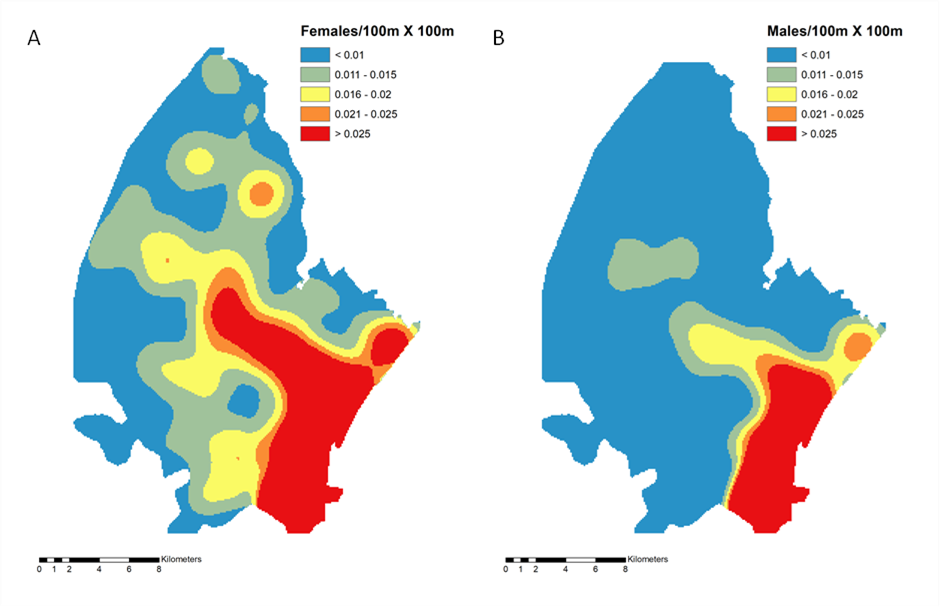

Supplement: S3 Fig — Density of in-migration into which external migrants from outside surveillance area moved by gender: (A) Females and (B) Males. Continuous surface maps of the density of external in-migrants were generated using a moving two-dimensional Gaussian kernel of 3 km search radius to produce robust density estimates that vary across continuous geographical space to generate a grid of 100m x 100m pixels. The size of the kernel was determined from the results of previous work [33]. The kernel moves systematically across the map and measures spatial variation in the density of in-migrants across the surveillance area. (TIF) [file pgph.0000055.s003.tif]
