# Supplementary material for: The geography and inter-community configuration of new sexual partnership formation in a rural South African population over fourteen years (2003–2016)
Source: PLOS Glob Public Health. 2022 Mar 9;2(3):e0000055. doi: 10.1371/journal.pgph.0000055 (PMC10022024; doi:10.1371/journal.pgph.0000055)
Supplement: Attachment — Submitted filename: Migration-Topology-PLoS Global Public Health_RL_20210927.docx [file pgph.0000055.s004.docx]

**RESPONSE TO REVIEWERS**

**Journal Requirements:**

1. You indicated that you had ethical approval for your study. We note that written informed consent was obtained for HIV testing. In your Methods section, please state whether consent was obtained for participation in this study, including all procedures and data collection and analysis.

In addition, please ensure you have also stated whether you obtained consent from parents or guardians of the minors included in the study or whether the research ethics committee or IRB specifically waived the need for their consent.

We now include the following statement:

Line 99-103: *“Written informed consent was also obtained for participation in the surveillance including all study procedures, data collection, and analysis from all individuals ≥18 years, whereas parental/guardian consent with child assent was sought for eligible individuals aged 15 to 17 years.”*

2. We ask that a manuscript source file is provided at Revision. Please upload your manuscript file as a .doc, .docx, .rtf or .tex. If you are providing a .tex file, please upload it under the item type ‘LaTeX Source File’ and leave your .pdf version as the item type ‘Manuscript’.

We have uploaded our manuscript file as a word document.

3. Please amend your detailed Financial Disclosure statement. This is published with the article, therefore should be completed in full sentences and contain the exact wording you wish to be published.

i). Please include all sources of funding (financial or material support) for your study. List the grants (with grant number) or organizations (with url) that supported your study, including funding received from your institution. 
ii). State the initials, alongside each funding source, of each author to receive each grant.
iii). State what role the funders took in the study. If the funders had no role in your study, please state: “The funders had no role in study design, data collection and analysis, decision to publish, or preparation of the manuscript.”
iv). If any authors received a salary from any of your funders, please state which authors and which funders.

We have updated the Financial Disclosure statement with detailed information as suggested.

This work was supported by three National Institute of Health (NIH) grants (R01HD084233, R01AI124389, and R21TW011687 (Recipient: FCT)). The Africa Health Research Institute’s Demographic Surveillance Information System and Population Intervention Programme is funded by the Wellcome Trust (201433/Z/16/Z) and the South Africa Population Research Infrastructure Network (funded by the South African Department of Science and Technology and hosted by the South African Medical Research Council). The funders had no role in study design, data collection and analysis, decision to publish, or preparation of the manuscript.

4. Please ensure that the funders and grant numbers match between the Financial Disclosure field and the Funding Information tab in your submission form. Note that the funders must be provided in the same order in both places as well.

We have checked that the funders and grant numbers match between the Financial Disclosure field and the Funding Information tab.

5. Please provide separate figure files in .tif or .eps format only, and remove any figures embedded in your manuscript file. If you are using LaTeX, you do not need to remove embedded figures.

For more information about figure files please see our guidelines: <https://journals.plos.org/globalpublichealth/s/figures>

We have provided separate figure files.

6. We notice that your supplementary figures are included in the manuscript file. Please remove them and upload them  with the file type 'Supporting Information'. Please ensure that all Supporting Information files are included correctly and that each one has a legend listed in the manuscript after the references list.

We have removed the supplementary figures from the main text and included them as separate supporting information documents.

7. Please provide us with a direct link to the base layer of the map used in Figures 1, 4, Supplementary Figures 1, 3 and ensure this location is also included in the figure legend.

Please kindly note that we did not use any base layer for the maps in Figures 1, 4, Supplementary Figures 1, and 3. As you can see in the figures, the background of the figures is blank. Please let us know if you have any further questions.

Please note that, because all PLOS articles are published under a CC BY license (creativecommons.org/licenses/by/4.0/), we cannot publish proprietary maps such as Google Maps, Mapquest or other copyrighted maps. If your map was obtained from a copyrighted source please amend the figure so that the base map used is from an openly available source.

Please note that only the following CC BY licences are compatible with PLOS licence: CC BY 4.0, CC BY 2.0 and CC BY 3.0, meanwhile such licences as CC BY-ND 3.0 and others are not compatible due to additional restrictions. If you are unsure whether you can use a map or not, please do reach out and we will be able to help you.

The following websites are good examples of where you can source open access or public domain maps:

* U.S. Geological Survey (USGS) - All maps are in the public domain. (http://www.usgs.gov)

* PlaniGlobe - All maps are published under a Creative Commons license so please cite “PlaniGlobe, http://www.planiglobe.com, CC BY 2.0” in the image credit after the caption. (http://www.planiglobe.com/?lang=enl)

* Natural Earth - All maps are public domain. (http://www.naturalearthdata.com/about/terms-of-use/)

Additional Editor Comments (if provided):

In light of the reviews, we would like to invite the resubmission of a significantly-revised version that takes into account the reviewers' comments. Another weakness of your manuscript is about statistical approaches and clustering method. More explanation or adapted references are needed.

Thank you for the comments. We have updated as following:

Line 154: *“… previously identified through Kulldorff spatial scan statistics analysis [34]…”*

Line 155-156: *“The methodology has been widely used in various public health research [36–39] and described in detail elsewhere [36].”*

Line 167-168: *“, and p-values are reported using a chi-square test of independence [41].”*

We thank the reviewers who provided very valuable comments. We have reviewed all the comments and revised our manuscript accordingly.

**Reviewers' comments:**

**Reviewer #1:** The paper presents important data on the geographic network structure of stable sexual relationships in rural KwaZulu-Natal, South Africa. While the methods, analysis, and conclusions are reasonable, I think readers would benefit from a more in-depth engagement with previous work on geospatial/network interventions and the implications these current findings have on this work. The integration of sexual network and geospatial data remains relatively limited but geographically targeted interventions have been discussed for many years. Given the high quality data used in the current manuscript, I think it would be beneficial to move beyond vague references to how these data support the notion of targeted interventions and try to propose ways in which this data could be specifically used to inform these efforts. For example, what do the data say about what such interventions should look like or what data would be needed to better inform the development of these interventions? A more thorough discussion of these topics would increase the likelihood of this manuscript leading to development and testing of the interventions proposed in the manuscript.

Thank you for the comment. We agree that further discussion regarding the implications of the study findings on future interventions would be informative. We have added the following:

Line 268-275: *“Several studies have proposed targeting resources and interventions to geographically clustered areas with high HIV prevalence and incidence [26,44,45] or populations at high risk of HIV infection [46]. Our study findings suggest that improving access to HIV prevention, testing, and care in this geographically clustered area could not only provide health benefits at the individual-level but also be an effective way to prevent HIV transmission by disrupting the connectivity of the entire contact network in the community and maximize resources needed to achieve epidemic control. So far, limited studies have tested the effectiveness of geographically targeted programs.”*

**Reviewer #2:** The authors present work looking at the spatial distribution of male-female partnerships in a demographic surveillance site in rural South Africa. Since the site is population-based, this represents a rare opportunity to understand relationship formation in relation to areas of high or low HIV prevalence.

While the approach appears sound and the conclusions are well justified from the results, there's a few areas where greater clarity could help to avoid confusion for the reader.

Major comments

1. p5, relationship information: I was wondering if the authors could provide more details on how the relationship information is collected. Was this from the individual survey of resident women in the household? If that's the case, what does the response rate look like over time? Is it the case that most women can be found for interview, and that most of them consent to the interview? Of those who do consent, do most also respond about their partnerships or are there refusals? This to me is crucial, as a key strength of the study is the population-based aspect, so it would strengthen the paper to provide greater details on the coverage of the partnership data.

Thank you for the comment. The information on marital status and sexual partnership was collected during household surveillance from all female household members aged ≥ 15 years. Participation in the household-level surveillance, including data collection on sexual partnership information, was extremely high (>98%), was extremely high (>98%). We have updated the relevant description in Methods as following:

Line 105-109: “*During household surveillance, detailed information on stable sexual partnerships, including the start and end dates of the relationships, was separately sought from all female household members aged ≥ 15 years. Participation in the household-level surveillance, including data collection on sexual partnership information, was extremely high (>98%) and remained stable over time [32].”*

2. p5, relationship information: It wasn't clear to me how the male partners were linked through surveillance? Was it through a matching approach or algorithm? More detail on this would also strengthen our understanding of how the relationship data was formed.

Thank you for the comment. As described in line 105-114, we only included stable sexual partnerships. By definition, the male and female partners in a stable sexual relationship belonged to the same household. Therefore, we were able to obtain detailed information on both male and female partners during the household surveillance including their names and unique surveillance identifiers. We have updated the relevant description as following:

Line 114-116: *“Information on male partners registered in the same households was therefore also obtained during the household surveillance and linked using their individual identifiers, and a unique identifier was given to each stable sexual partnership.”*

3. p5, relationship information: I didn't quite follow how time was handled in the network formation. I see how time is used for other aspects of the analysis, but I didn't quite follow how the network was built with the time aspect. I do note that the authors discuss the limitation of not examining this in a longitudinal way, so I'm more just unsure how the authors are considering time periods in terms of the network at time of partnership formation.

For the spatial network configuration, we used the locations at the start of partnership formation in a cross-sectional way. We have updated as following:

Line 139-141: “*Using the geographical location of individuals at the start of partnership formation, we conducted several network analyses to identify network structure characteristics.”*

Line 169-171: “*For the stable sexual partnerships in which partners were both residents within the study area, each partner’s homestead geographical location was linked to the other partner’s location at the start of sexual partnership.”*

4. p5, p6 network configuration: I was wondering for the two network measures (node degree and eigenvector centrality) if the authors could provide a bit more guidance for the reader on the interpretation? For readers not familiar with these measures, perhaps a small example network where the authors present these two measures and what they mean would make it easier for the reader to then interpret the main results.

Thank you for comment. We agree that more description would be helpful for the readers to interpret our results. We have updated as following:

Line 141-143: “*First, we assessed the association between the node degree (i.e., the number of connections that a node has to other nodes in the network – the higher the degree is, the more connected the node is).”*

Line 145-148: “*Second, we calculated the eigenvector centrality of the node, which is a measure of its influence in the network based on the degree of being linked to other highly connected nodes (i.e., the higher eigenvector score is, the more connected the node is to other nodes which also have high eigenvector scores themselves).*

Minor comments

5. p7, line 201: The authors note nodes with no connections were excluded, I was wondering if they could report how many there were that were excluded?

Thank you for comment. We have updated as following:

Line 214: “Excluding the 10 nodes which had no connections, node degree …”
